# Medical Classes

**Published:** 1845-03

**Authors:** 


### MEDICAL CLASSES.

From various sources, we have gleaned the following particulars of the number in attendance on the lectures the past winter, at different Colleges: viz—

| | |
|---|---|
| University of Pennsylvania | 446 |
| Jefferson Medical College | 409 |
| Louisville Medical Institute | 286 |
| College of Physicians and Surgeons, N. Y. | 190 |
| Geneva College | 183 |
| Harvard University, (Boston) | 157 |
| Berkshire Medical Institution | 145 |
| Willoughby Medical College | 126 |

From the other schools we have received no catalogues, or other authentic information of the number of their classes.

